# 3D melanoma spheroid model for the development of positronium biomarkers

**DOI:** 10.1038/s41598-023-34571-4

**Published:** 2023-05-11

**Authors:** Hanieh Karimi, Paweł Moskal, Agata Żak, Ewa Ł. Stępień

**Affiliations:** 1 Department of Medical Physics, M. Smoluchowski Institute of Physics, Faculty of Physics, Astronomy and Applied Computer Science, Jagiellonian University, Łojasiewicza 11 Street, 30-348 Kraków, Poland; 2 Department of Biochemistry, University of Missouri, Columbia, USA; 3 Department of Experimental Particle Physics and Applications, M. Smoluchowski Institute of Physics, Faculty of Physics, Astronomy and Applied Computer Science, Jagiellonian University, Kraków, Poland; 4 Center for Theranostics, Jagiellonian University, Kraków, Poland; 5 Faculty of Chemistry, Jagiellonian University, Kraków, Poland

**Keywords:** Nanoscale biophysics, Cellular imaging, Diagnostic markers, Biophysics

## Abstract

It was recently demonstrated that newly invented positronium imaging may be used for improving cancer diagnostics by providing additional information about tissue pathology with respect to the standardized uptake value currently available in positron emission tomography (PET). Positronium imaging utilizes the properties of positronium atoms, which are built from the electrons and positrons produced in the body during PET examinations. We hypothesized that positronium imaging would be sensitive to the in vitro discrimination of tumor-like three-dimensional structures (spheroids) built of melanoma cell lines with different cancer activities and biological properties. The lifetime of ortho-positronium (*o*-Ps) was evaluated in melanoma spheroids from two cell lines (WM266-4 and WM115) differing in the stage of malignancy. Additionally, we considered parameters such as the cell number, spheroid size and melanoma malignancy to evaluate their relationship with the *o*-Ps lifetime. We demonstrate pilot results for *o*-Ps lifetime measurement in extracellular matrix-free spheroids. With the statistical significance of two standard deviations, we demonstrated that the higher the degree of malignancy and the rate of proliferation of neoplastic cells, the shorter the lifetime of ortho-positronium. In particular, we observed the following indications encouraging further research: (i) WM266-4 spheroids characterized by a higher proliferation rate and malignancy showed a shorter *o*-Ps lifetime than WM115 spheroids characterized by a lower growth rate. (ii) Both cell lines showed a decrease in the lifetime of *o*-Ps after spheroid generation on day 8 compared to day 4 in culture, and the mean *o*-Ps lifetime was longer for spheroids formed from WM115 cells than for those formed from WM266-4 cells, regardless of spheroid age. The results of this study revealed that positronium is a promising biomarker that may be applied in PET diagnostics for the assessment of the degree of cancer malignancy.

## Introduction

Over the past few decades, three-dimensional (3D) cell cultures have been widely used as in vitro models, which can bridge the gap between in vitro and in vivo cell conditions^1^. The comparison of the 3D cell culture to a cell monolayer revealed some specific physiological and morphological characteristics, such as cell-to-cell and cell–matrix interactions, cell signaling, proliferation and necrosis. Unlike a monolayer cell culture, a 3D spheroid is an appropriate model to mimic the real tumor cell environment and nutrient diffusion rate between cells. Multicellular tumor spheroids allow us to study the biochemical mechanism of cell growth, enzymatic reactions, and various treatment modalities^2–5^.

**Figure 1:**
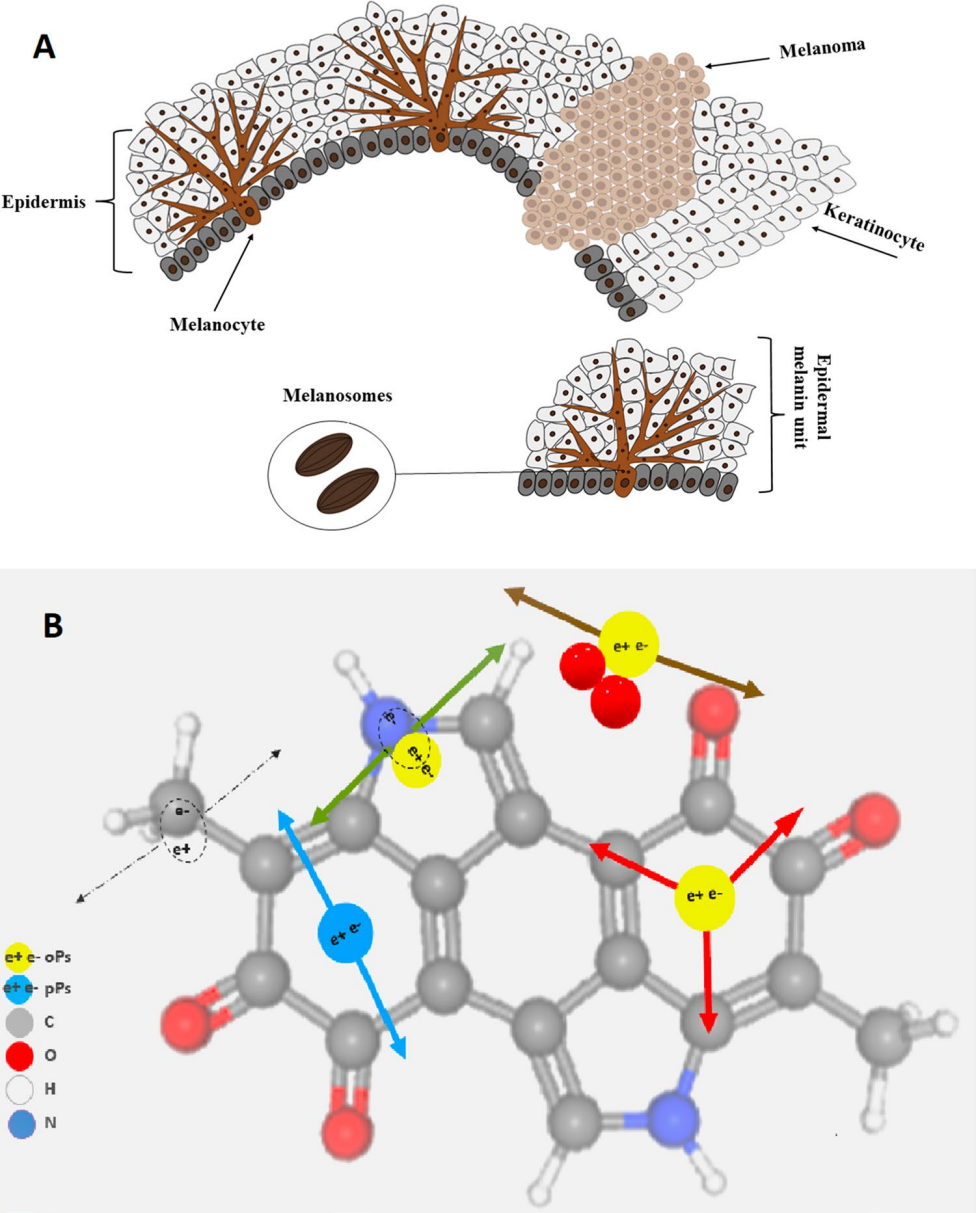
Schematic view of melanocytes and the formation of a melanoma lesion in the epidermal layer. (**A**) Normal melanocytes have a regular dendritic shape and form many branching processes extending between numerous keratinocytes (left). One melanocyte reaches between 36 and 40 keratinocytes, forming an epidermal melanin unit (EMU), with a balanced proportion of one melanocyte to each of 8–10 keratinocytes in the basal layer of the epidermis. In a melanoma lesion (right), melanocytes lose their dendricity and become malignant and amoeboid in shape, changing their cell–cell contacts by expressing different adhesion molecules (*N*-cadherin instead of *E*-cadherin expressed by melanocytes)^7^. Melanosomes store the melanin granules produced by melanocytes, which can be distributed among surrounding keratinocytes to protect them from UV radiation damage. (**B**) Pictorial illustrations of positron annihilations in the melanin molecule. Carbon, oxygen, hydrogen, nitrogen, *p*-Ps and *o*-Ps atoms are indicated in colours explained in the legend. Dashed arrows indicate photons from direct electron–positron annihilation. Red arrows indicate photons from *o*-Ps atom self-annihilation. Blue arrows show photons from *p*-Ps self-annihilation. Green and brown arrows indicate *o*-Ps decay via the pick-off and conversion processes, respectively.

Melanoma is a prevalent type of skin cancer that has been categorized as one of the most lethal cancers. Melanoma, as the most lethal cancer, is a multifactorial disease in which both genetic susceptibility and environmental exposure, predominantly to ultraviolet light, play important roles^6^. The appointed environmental risk factors for melanoma cancer are exposure to solar ultraviolet radiation (UVB) and sunburns (UVA). UVB irradiation causes direct DNA damage, which leads to DNA strand breaks. UVB also promotes melanoma cell survival, angiogenesis, and invasion due to increases in the penetration of macrophages and neutrophils into skin cells. UVA causes DNA damage through the production of free radicals, which induce oxidative stress in melanocytes. The risk for melanoma can also be associated with inherited mutations and somatic mutations, but the genetic tendency is responsible for only a small number of cases. As the effectiveness of melanoma treatment at advanced stages is low, there is a constant need for the development of new targeted therapies, immunotherapy, and combined therapies^7^ (Fig. 1).

Although numerous studies have been dedicated to diagnosing melanoma in the early stages, more investigation and follow-up are needed. The rate of metastasis in patients due to melanoma is high, with 350,000 new cases per year, and the number of new cases is significantly increasing each year^8–10^.

In this study, a 3D spheroid model of two melanoma cell lines, WM266-4 and WM115, with different stages of malignancy was evaluated. The melanoma cell lines WM266-4 and WM115 have various biological properties that make them different in proliferation, migration rate, and cell size. The activation of advanced glycation end products (AGEs), which are produced during the combination of lipids and proteins with sugar in human metabolism, and proteins such as the receptor for AGEs (*RAGE*) and c-Jun N-terminal kinases (*JNK*) in the WM266-4 line makes this cell line more invasive than the WM115 line^11^. Moreover, the expression of the *SLC7A5* gene (variant of Large Amino-acid Transporter 1) in WM115 is higher than in WM266-4, in contrast to the expression of the *SLC7A8 * gene, which is lower in WM115 than in WM266-4^12^. These genes are involved in amino acid import and are essential for cell pigmentation, and the WM115 cell line is darker than the WM266-4 cell line.

In this study, we hypothesized that the difference between the grade of malignancy of WM115 and WM266-4 melanoma cancers present at the level of cell physiology can be probed by positronium biomarkers. A positronium is an exotic atom built of positrons and electrons^13^. The positronium is also formed in the intramolecular spaces during PET diagnosis^13^. In the tissue, the positronium may be formed and trapped in the free voids of the intramolecular spaces, as shown pictorially in Fig. 1^14^. The positron emitted from the radionuclide (e.g., ^18^F in the PET diagnosis or ^22^Na in the typical Positron Annihilation Lifetime Spectroscopy (PALS) experiments) penetrates the object, and after losing the energy, annihilates with an electron from the molecules constituting the cells. The electron–positron annihilation into photons may occur directly (e + e− → photons (black dashed arrows in Fig. 1B)) or *via*a positronium atom [e + e− → positronium → photons (solid arrows in Fig. 1B)]. In a quarter of cases, the positronium is formed as a short-lived (125 ps) state called the para-positronium (*p*-Ps), and in three-quarters of cases, it is formed as a long-lived (142 ns) ortho-positronium (*o*-Ps). Para-positronium (indicated in blue in Fig. 1B) decays predominantly into two photons (blue arrows), and ortho-positronium decays in vacuum predominantly into three photons (red arrows). However, in the intramolecular voids, ortho-positronium undergoes processes such as pick-off (the positron from *o*-Ps annihilates into two photons (green arrows) with an electron from the surrounding molecule) and conversion into para-positronium via interaction with molecules such as oxygen molecules. The resulting para-positronium decays into two photons (brown arrows). The range of the *o*-Ps mean lifetime variation is significant, and it changes from 142 ns in vacuum to 1.8 ns in water^15,16^. Therefore, the ortho-positronium lifetime depends strongly on the molecular environment: the nanomolecular structure and concentration of the bioactive molecules^13–19^. The properties of positronium in biological samples have been scarcely studied thus far. Only recently were the first studies of positronium in 3D cell structures performed by culturing cells on collagen scaffolds^20^. Other studies on skin cancer cells (basal and squamous cell carcinomas) were performed using a low-energy positron beam that limited skin penetration^21–23^.

The first in vitro research on the positronium lifetime in tissues from patients indicated promising results in view of the application of positronium as a biomarker for the diagnosis of uterine cancer and myxoma cancer and as a biomarker of hypoxia^24–27^. Recently, positronium was proposed as a novel biomarker for the in vivo assessment of tissue pathology^13,25^ that can be imaged using a newly developed positronium imaging method^15,19,26,28^ when applying prompt photon radionuclides^28–31^ and high-sensitivity PET scanners^28^. Moreover, the advent of total-body PET systems characterized by high sensitivity^32–35^ will enable the simultaneous application of positronium imaging to standard metabolic imaging during positron emission tomography^29,36,37^.

The aim of this study was to determine whether positronium imaging would be sensitive to the in vitro discrimination of tumour-like three-dimensional structures (spheroids) built from melanoma cell lines with different cancer activities and biological properties.

## Methods

### Cell culture

Two melanoma cell lines, WM266-4, a human malignant melanoma cancer cell line, and WM115, a cell line from a primary melanoma, were purchased from ESTDAB Melanoma Cell Bank (Tübingen, Germany) and cultured as we previously described^38^. A Luna-II™ automated cell counter (Logos Biosystems, Inc.) was used to determine the cell counts and viability before cell seeding.

### Spheroid generation

Both cell lines, WM266-4 and WM115, were seeded in 5D spherical plates (5D sp5dplate, Kugelmeiers, Switzerland) to form spheroids^39^. The 5D microplate has 24 wells, 12 wells with nanocoated surface for spheroid formation, and 12 wells as a control. In our study, control wells were not used. Each well contains 750 microcavities (with 509 µm diameter and 320 µm depth) that are separated from each other by sharp borders, and these borders prevent cell migration from one microcavity to another; thus, 9000 spheroids with uniform shapes and diameters can be cultured in a single plate.

For cell seeding, 0.5 ml of complete medium was added to each well. Then, an extra 0.5 ml medium including 1,125,000 cells/well (1500 cells/microwell) was added to each well of the plate. The cells in the Falcon tube were resuspended to distribute them in the whole medium and then added to the wells.

After cell seeding, plates were maintained in an incubator at 37 °C and 5% CO_2_ with humidity. The cell culture medium was renewed every day after the spheroids were formed. Two suitable time points of spheroid growth were chosen to measure the mean o-Ps lifetime. Spheroid morphology and proliferation rate were also determined under an optical microscope (Olympus, IX-LWPO, T2, Japan) on days 4 and 8 after cell seeding. Image analysis was conducted by ImageJ software.

### Viability test assay

**Figure 2:**
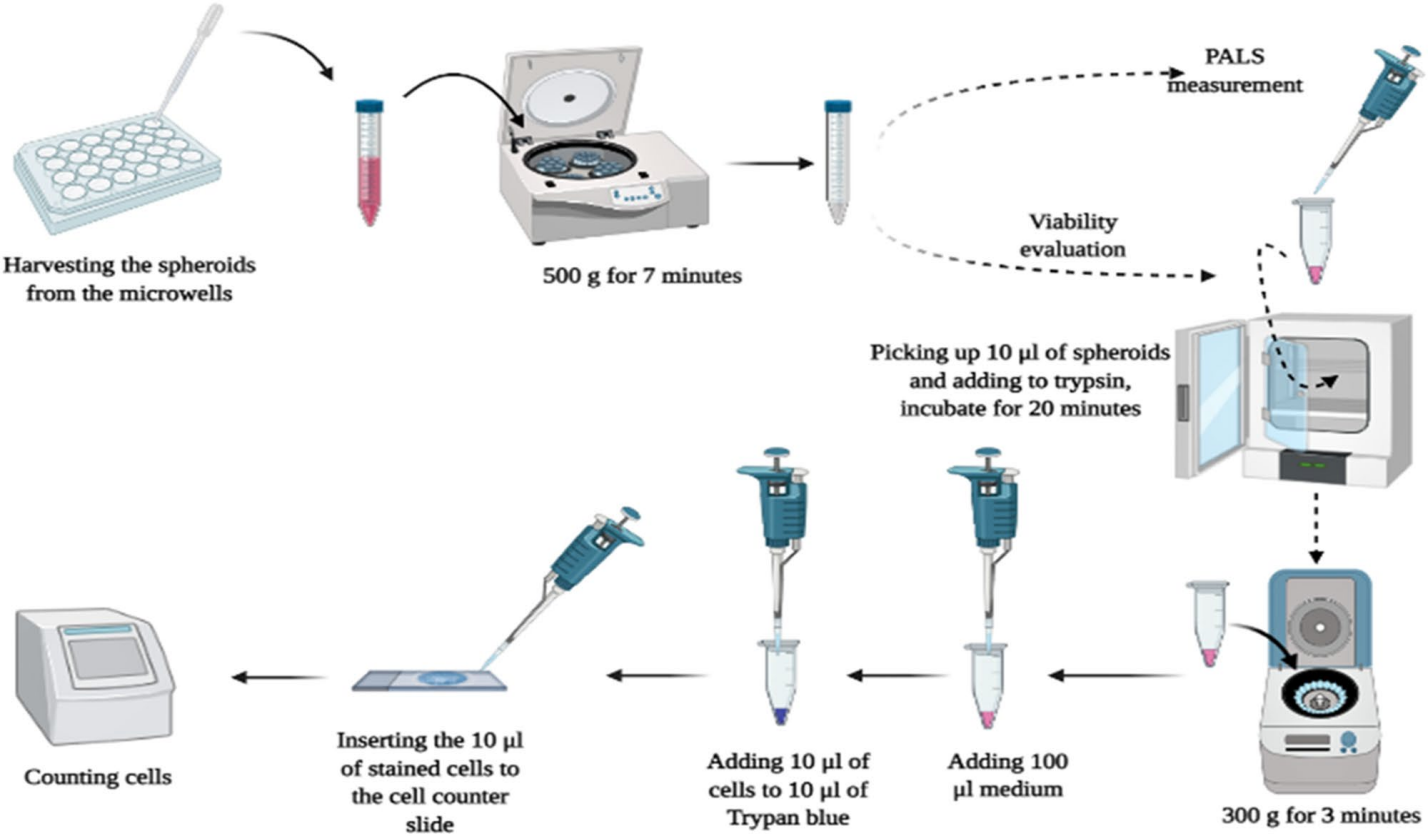
Workflow for investigation of spheroid viability after harvesting 3D spheroids from 5D microplates.

First, spheroids were dissociated to a single-cell suspension with trypsin/EDTA (Gibco, cat. no. 25200072, Waltham, MA USA). Then, 100 µl of trypsin/EDTA Spheroids was incubated for 10–20 min at 37 °C and pipetted several times to separate the cells. Then, the cells were centrifuged at 300*g* for 3 min, the supernatant was removed, and 100 µl of medium (Gibco, cat. no. 10010056, Waltham, MA, USA) was added to the cells and pipetted several times to separate the cells completely. In the final step, 10 µl of cells was added to 10 µl trypan blue and counted (the Luna-II™ cell counter, Logos Biosystems, Aligned Genetics, Inc). The workflow is shown in Fig. 2. Each viability test was performed on 4 different samples in each measurement under the same conditions.

### Estimation of cell size

After 15 min in the incubator, the cells were checked to ensure that there were no aggregates. After the viability test using the cell counter, the cell cluster map was evaluated to determine the percentage of clusters. In our experiments, cluster maps showed a high percentage of dissociation, above 90%. Afterward, the size of cells for both cell lines in 3D shape was checked by cell counter and microscopic investigations. For estimation of the cell size,

A Luna II ™ cell counter was used. Regarding the histogram of size distribution, the mean diameter of cells was estimated. For comparison, the size of cells in 2D cultures was also determined. For this purpose, spheroids were removed from each well using a 3 ml pipette with a large bore to avoid destroying the spheroid structure. Then, spheroids were poured onto a 15 ml Falcon tube and centrifuged at 500*g* for 7 min. In the next step, the supernatant was removed, and 1 ml fresh medium was added to the spheroids.

### Glucose distribution

To evaluate glucose uptake, a fluorescence-emitting d-glucose probe 2-NBDG (2-(*N*-(7-nitrobenz-2-oxa-1,3-diazol-4-yl)amino)-2-deoxyglucose) (Thermo Fisher Scientific, cat. no N13195) was used. To assess the oxygen uptake rate distribution in the spheroids, WM266-4 and WM115 cells were seeded at densities of 1000 and 2000 cells/drop. On days 4 and 8, the spheroids were transferred to glass-bottom dishes, and 20 μl of 2-NBDG (200 μM) was added to each spheroid. The spheroids were incubated at 37 °C for 45 min. Finally, spheroids were washed with PBS, and spheroid images were taken with a Nikon Eclipse Ti-E microscope coupled to an A1 scanning confocal system (Nikon, Japan) at a 494 nm/551 nm fluorescent wavelength.

### Hypoxia distribution in spheroids

Oxygen uptake rate distribution was determined using a hypoxia kit, Image-IT™ Green Hypoxia Reagent (Thermo Fisher Scientific, cat. no I14834). To assess the oxygen uptake rate distribution in the spheroids, WM266-4 and WM115 cells were seeded at densities of 1000 and 2000 cells/drop. On days 4 and 8, the spheroids were transferred to glass-bottom dishes, and 20 μl of Image-IT™ Hypoxia Reagent (10 μM) was added to each spheroid. The spheroids were incubated at 37 °C for 1 h. The hypoxia dye was exchanged with fresh growth medium, and the spheroids were placed again in a cell culture incubator for the next 4 h. Spheroid images were taken with a Nikon Eclipse Ti-E microscope coupled to an A1 scanning confocal system (Nikon, Japan) at a 488 nm/520 nm fluorescent wavelength.

### Positron annihilation lifetime spectroscopy in 3D spheroids

To obtain reliable and precise results of the positronium lifetime in spheroids, we used spheroids without any medium, supernatant, or chemical compounds. In this method, harvested spheroids from the microplates were prepared for positronium lifetime measurement after centrifugation and removal of the medium completely from the spheroids.

**Figure 3:**
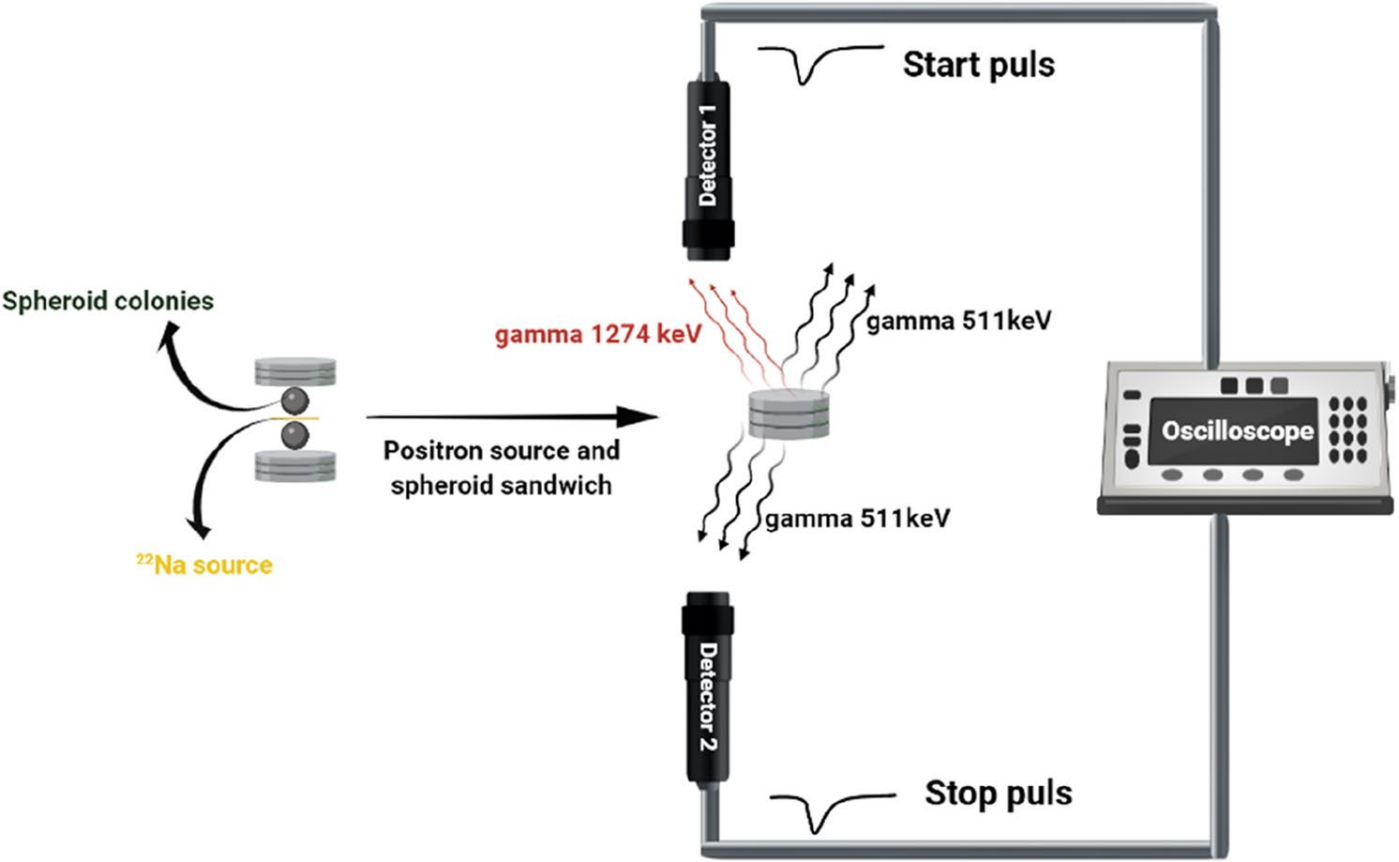
Spheroids surrounding the ^22^Na source are located in the dedicated chamber between two BaF_2_ detectors. Spheroids are adjacent to the ^22^Na source, and there is no space or bubble between them. For each measurement, 10^6^ events with the coincident registration of 511 keV photons and 1274 keV photons were collected.

The positronium lifetime was measured using a spectrometer consisting of two vertically arranged BaF_2_ plastic scintillators (SCIONIX, Holland) and two photomultipliers with serial numbers SBO696 and SBO697 (Hamamatsu, Japan) powered by a high voltage power supply (CAEN SY4527). The signals from photomultipliers were read out and analyzed by the 6000A data analyzer oscilloscope (Le Croy). Spheroids were irradiated by positrons emitted from the ^22^Na radionuclide (with an activity of approximately 1 MBq) placed in Kapton foil. A dedicated aluminum chamber was used as a container for spheroids, and a holder was connected to a heater to keep the cells at 37 °C. The measurement setup is shown pictorially in Fig. 3.

**Figure 4:**
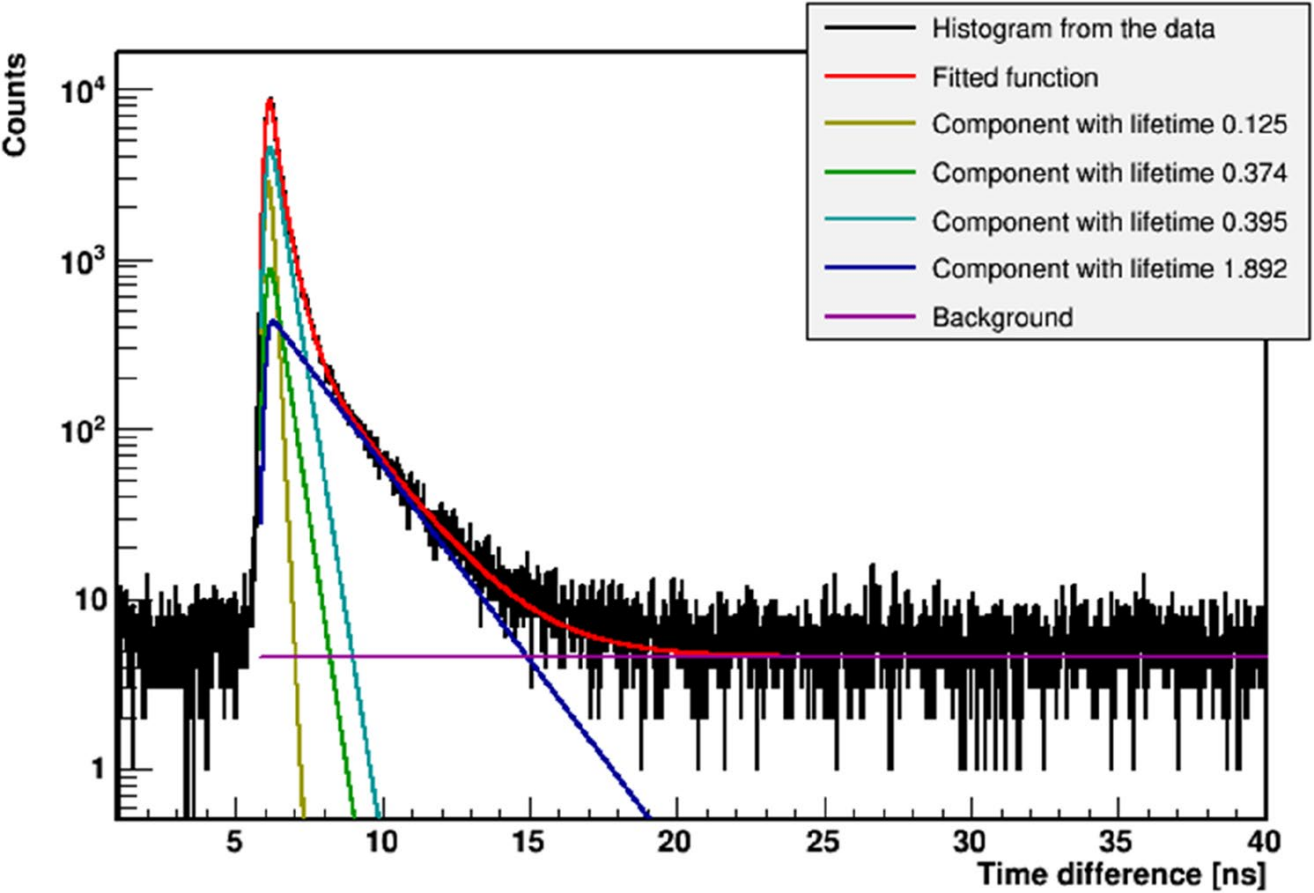
Example positronium lifetime spectrum. Experimental data (black histogram) with superimposed histograms resulting from the fit of the sum of the exponential function convoluted with the detector resolution, performed by means of the PALS Avalanche program^25,40^. The first component (yellow line) shows the contribution from *p-*Ps (mean lifetime: 0.125 ns), the second component (green line) originates from annihilations in the source (Kapton foil) (0.374 ns), the third component (light blue) shows the free annihilation lifetime (0.395 ns), and the fourth component (dark blue) illustrates the contribution from *o-*Ps. The sum of all contributions resulting from the fit is shown as a red curve.

The ^22^Na radionuclide after emission of the positron transforms to the excited state of the ^22^Ne isotope that deexcites (on average after 2.6 ps) via emission of the 1274 keV gamma quantum^29,30^. The positron loses energy while passing through the cells and eventually annihilates with the electron into two back-to-back 511 keV gamma quanta. Positron–electron annihilation may proceed directly or via creation of the positronium. The time between the emission of the positron and its annihilation is measured by the registration of the 1274 keV deexcitation photon and one of the 511 keV annihilation photons. The sample with the source is positioned in a way (see Fig. 3) that enables coincident registration of 1274 keV gamma and one 511 keV photon, and it prevents the coincident registration of both 511 keV photons flying back-to-back. An exemplary lifetime spectrum determined as a result of the measurement is shown in Fig. 4.

Before each measurement, the system was calibrated using an empty chamber including only the ^22^Na source. To perform the measurement, spheroids were transferred from the centrifuge tube to the chamber by using a scalper, and ^22^Na radioactive sources were placed between the samples. Then, the spheroid sample was placed adjacent to the source, with no air between the source and spheroids. Then, the chamber was placed inside a holder that was connected to a heater to keep the cells at 37 °C. Finally, the holder was located between two BaF_2_ detectors, as indicated in Fig. 3.

The lifetime of positronium in spheroids at two different time points, the 4th and 8th days after seeding cells, was evaluated based on the 10^6^ events collected for each studied case. In this study, only spheroids without medium, water, and any chemicals were evaluated.

The positronium lifetime was extracted from each recorded spectrum by fitting the sum of four exponential functions convoluted with the detector resolution function^40,41^. The exemplary spectrum with the result of the fit is shown in Fig. 4. The fitted components correspond to the decay of para-positronium (yellow curve), direct annihilation in the source/Kapton foil (green curve), and annihilation of ortho-positronium (dark blue). The full experiment was repeated three times.

## Results

### Spheroid morphology and proliferation rate

**Figure 5:**
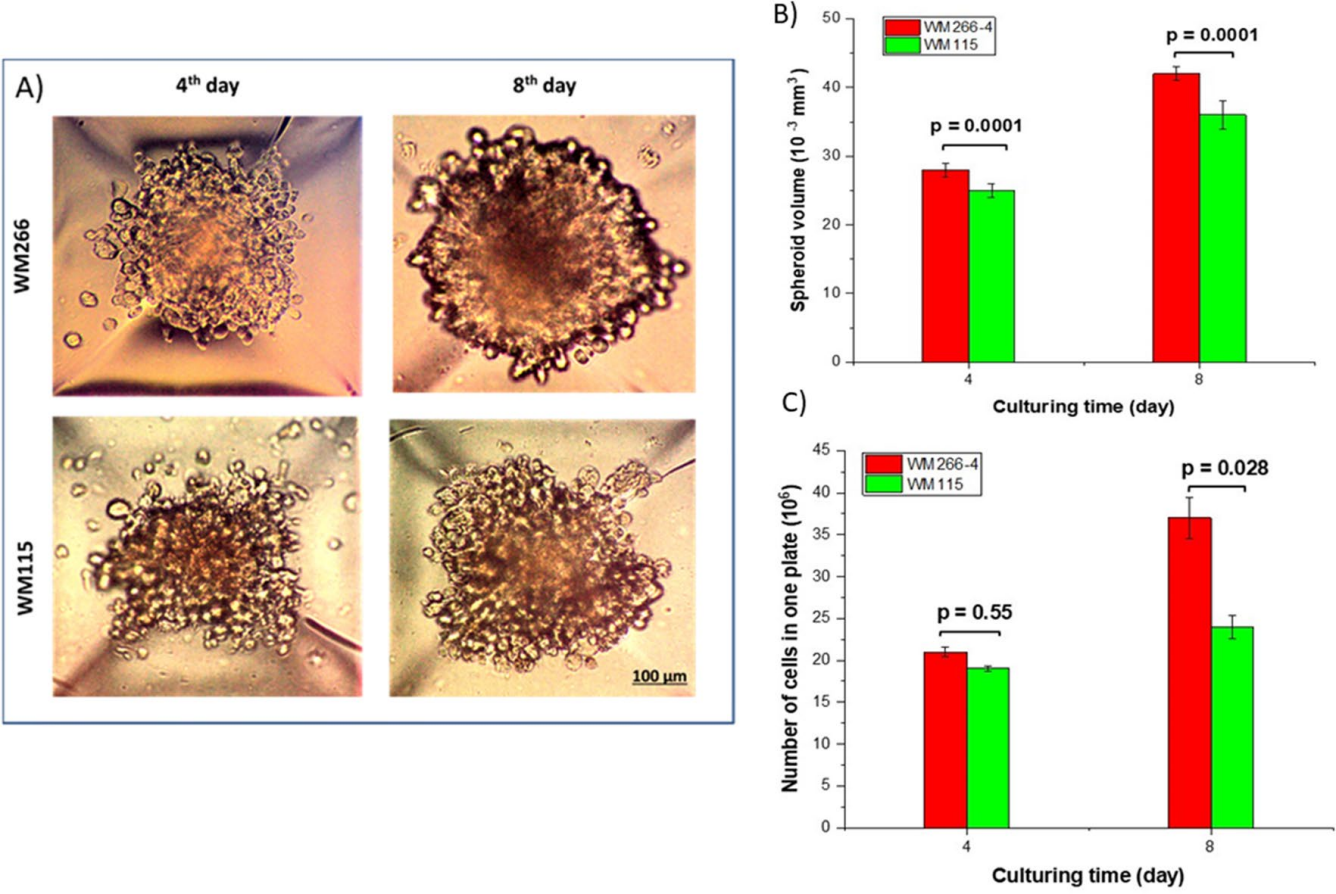
Comparison of spheroids from different melanoma cell lines. (**A**) Microscopic images of two different cell lines, WM266-4 and WM115, on days 4 and 8 of culturing. The density and circularity of spheroids increased during the culture time. (**B**) As a function of culturing time, WM266-4 spheroids showed a higher volume than WM115 spheroids. (**C**) The number of cells in the plate was increased during the culture time for both cell lines. The number of cells in one plate was calculated using the Luna II cell counter after spheroid harvesting.

The exemplary spheroids grown in the microplate are shown in Fig. 5. The WM266-4 cell line formed more spherical and concentrated spheroids than the WM115 cell line, as previously reported^38^. Spheroids in both cell lines showed an increase in their size and circularity over time. Generally, the rate of growth and size of spheroids depend on the size of cavities and the type of plates that have been seeded. The larger the microcavities are, the larger the formed spheroids⁠. Although the size of spheroids can be controlled by initial culture seeding, the scales of microwells can also affect the spheroid diameter^42^.

Figure 5B,C shows that WM266-4 and WM115 spheroids grew over time. WM266-4 spheroids exhibited a faster proliferation rate than WM115 spheroids, which returned to their malignant characteristics.

Figure 5B shows that there was an increase in the number of cells from cell seeding until the 8th day after culturing. The division rate of WM266-4 cells in spheroids was higher than that of WM115 cells. WM266-4 resulted in 1.5- and 2.74-fold increases in cell number after 4 and 8 days, respectively. The number of cells in WM115 spheroids increased 1.4- and 1.7-fold after 4 and 8 days, respectively. A total of 36,000 spheroids were used in each positronium lifetime measurement.

### Spheroid cell characterization

The WM266-4 spheroids comprised cells with a diameter of 15.70 ± 0.10 μm at day 4 after cell seeding and 15.92 ± 0.08 μm at day 8, while the WM155 spheroid cell diameter was equal to 16.66 ± 0.20 μm at day 4 and 17.28 ± 0.25 μm on day 8. The mean size of stained cells in 2D cell culture was smaller than the size of cells from the 3D spheroids, 14.65 ± 0.09 μm and 16.27 ± 0.10 μm for WM266-4 and WM115, respectively.

The number of cells in both cell lines increased over time. The observed growth was faster for the WM266-4 cell line than for the WM115 cell line. WM115 has a longer doubling time than WM266-4, approximately 7.5 and 6 days, respectively, which means that cells of the WM115 line spend more time in their cell cycle than cells from the WM266-4 line. The doubling time (DT) of spheroids is usually characterized as the real tumor doubling time and is calculated by using spheroid volume via:

$$DT=\frac{t.ln2}{ln\frac{{V}_{2}}{{V}_{1}}},$$

where V_1_ and V_2_ are spheroid volumes at times t_1_ and t_2_ = t_1_ + t after seeding, respectively^43–45^.

### Determination of glucose distribution

**Figure 6:**
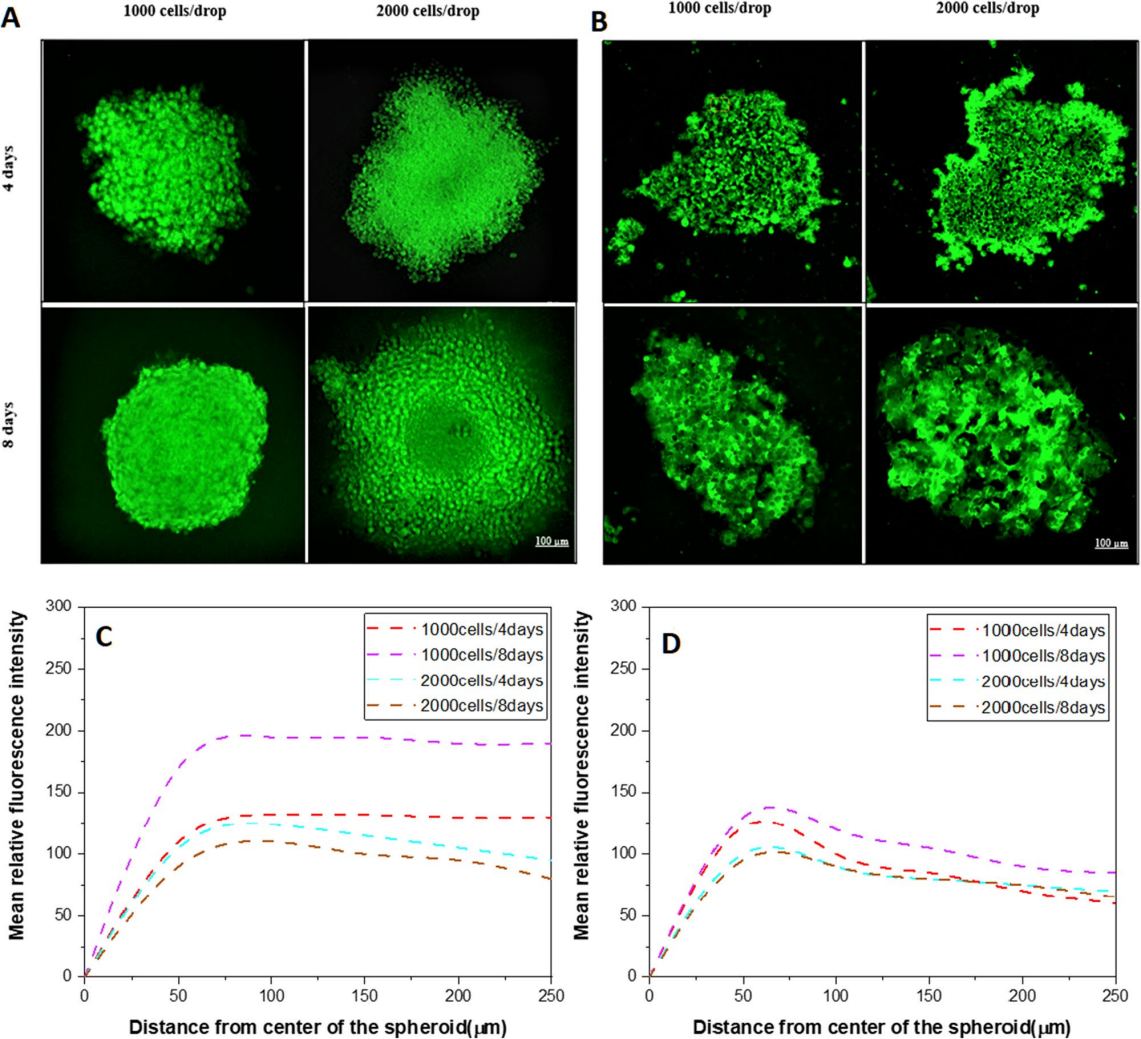
Determination of glucose distribution in melanoma spheroids. Glucose concentration is observed equally distributed in the proliferation rim in WM266-4 spheroids (**A**) and more dispersed WM115 cell lines (**B**) with significant differences in the larger and older spheroids. Plot of glucose distribution in WM266-4 (**C**) and WM115 (**D**) spheroids at two different culture time points as assessed with the 2-NBDG probe.

According to the fluorescence intensity analysis (Fig. 6), the region between 100 and 200 µm from the center of a spheroid was considered the outer layer, also called the proliferation rim, and the region between 50 and 100 µm of the inner part of the spheroid was considered as a necrotic core. However, the size of different layers of a spheroid depends on the size of the spheroid. Fluorescence intensity after 4 days in spheroids obtained from an initial number of 1000 cells at the edge of the WM266-4 spheroids was not significantly higher than that for WM115 (p = 0.20), while in the deeper region of spheroids, a higher intensity of glucose uptake was observed in WM266-4 spheroids than in WM115 spheroids (p = 0.00001). Larger spheroids formed from 2000 cells showed higher glucose uptake after 4 days, and the intensity was significantly higher in the proliferation rim (p = 0.04) and in the core area in WM266-4 spheroids vs. WM115 spheroids (p = 0.0018). On day 8, WM266-4 and WM115 spheroids showed a significant difference in the intensity of glucose uptake in the proliferation rim (p = 0.007) but not in the core (p = 0.064) (Fig. 6).

### Determination of the hypoxic region

**Figure 7:**
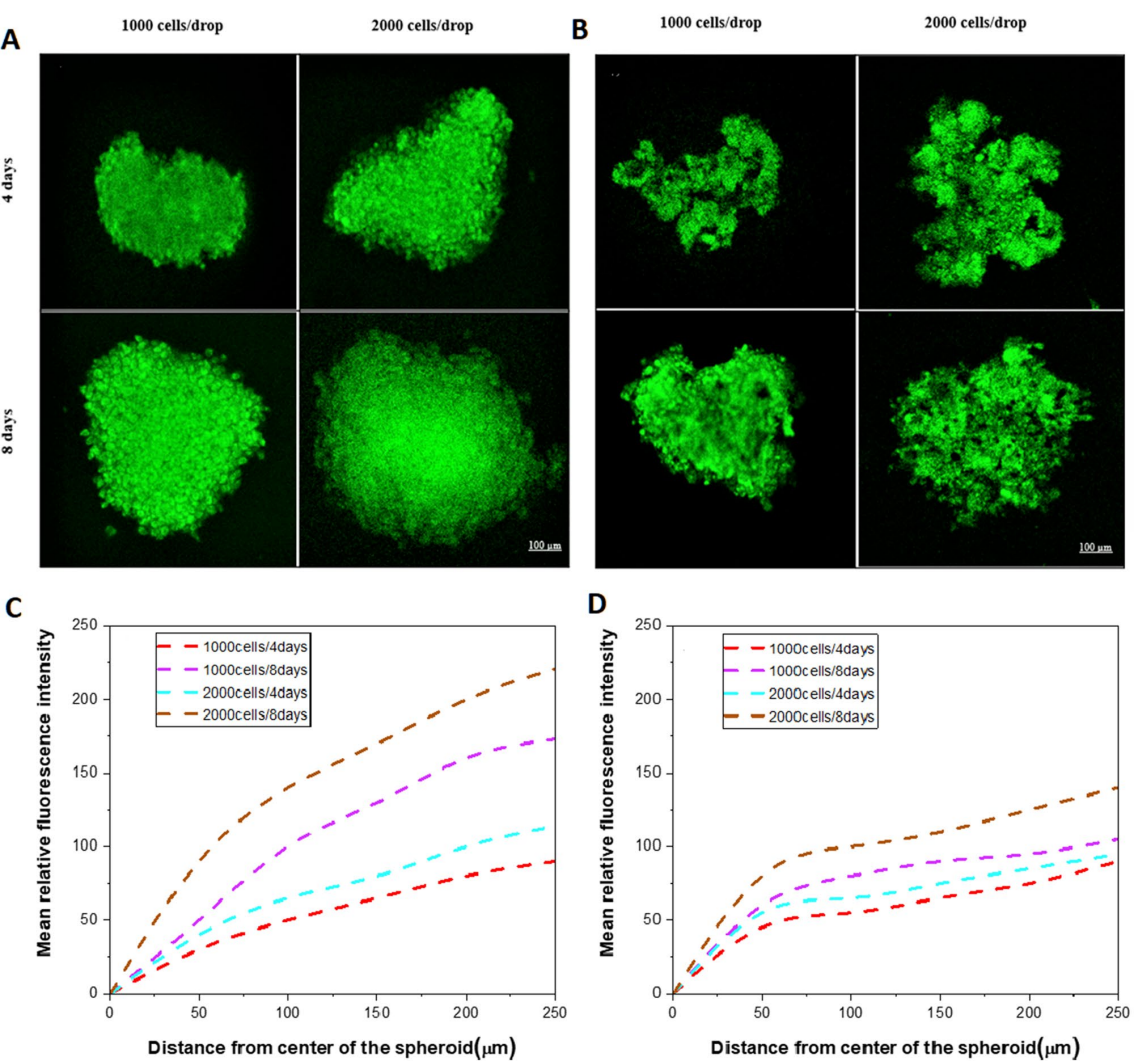
Determination of hypoxia progression in melanoma spheroids. The hypoxic region is observed in the center of spheroids in (**A**) WM266-4 and (**B**) WM115 cell lines with significant differences in the larger and older spheroids. Plot of hypoxia distribution in WM266-4 (**C**) and WM115 (**D**) spheroids at two different culture time points as assessed with the Image-IT™ Green Hypoxia probe.

On day 4 of culture, the progression of hypoxia measured as fluorescence intensity in spheroids with an initial cell number of 1000 was not significantly different (p > 0.05), while on day 8, the degree of hypoxia in the center of WM266-4 spheroids was significantly higher than that of WM115 spheroids (p = 0.0001). In the larger spheroids during culture, the degree of hypoxia in WM266-4 spheroids was significantly higher (p < 0.001) than that in WM115 spheroids, while at day 8, WM266-4 and WM115 spheroids showed a significant difference in the progression of hypoxia in their center (p = 0.00048) but not in the outer part (p = 0.24) (Fig. 7).

### The lifetime of ortho-positronium

The mean lifetime of ortho-positronium atoms in the spheroids was established for two different cell lines characterized by different degrees of malignancy: WM115 and WM266-4. Both measurements were performed for spheroids at two different time points: on days 4 and 8 after seeding. The viability tests were evaluated before and after each PALS measurement and showed that spheroid viability remained constant above 85% until the 8th day. Thus, the spheroids were in good and stable condition during the experiment.

The measurement of the positronium lifetime was repeated three times. In each measurement, spheroids were harvested from the plate and placed inside the chamber.

**Figure 8:**
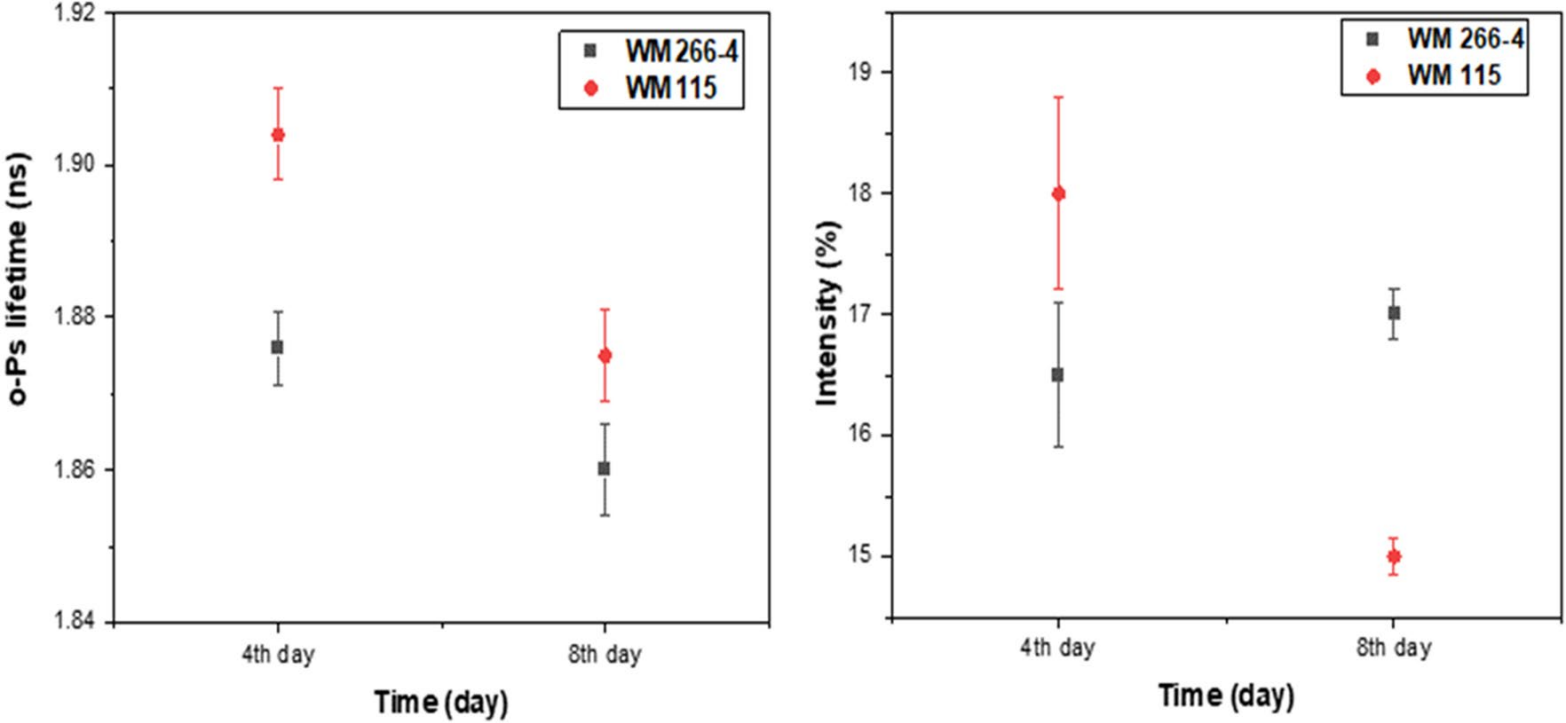
Comparison of the mean *o*-Ps lifetime and intensity for different melanoma cell lines. (Left) The lifetime of o-Ps in WM266-4 (black squares) and WM115 (red dots) spheroids in two different ages, 4 and 8 days after cell seeding. (Right) Intensity of o-Ps production in WM266-4 spheroids (black squares) and WM115 spheroids (red dots) as a function of time. To compare the mean *o*-Ps in relation to spheroid metabolism, please see supplementary file 1.

Figure 8 demonstrates the results of the mean *o*-Ps lifetime and distribution in 3D melanoma spheroids with different malignancy levels at two different ages.

The obtained results given in Table 1 show that WM266-4 spheroids with more malignancy, proliferation rate, and high concentration of cells in spheroids have a shorter ortho-positronium mean lifetime than WM115 cells, therefore, smaller free intermolecular voids, than WM115 cells from the primary tumor with less division rate and lower concentration of cells over time. Although both cell lines show a gradual decrease in the lifetime of *o*-Ps during the time of culturing, *o*-Ps show a longer lifetime in WM115 than in WM266-4 spheroids. The intensity in WM266-4 spheroids remained almost constant while WM115 spheroids present a decrease in the intensity of < 3SD >, which can be considered as changes at the molecular level in WM115 spheroids cells.

**Table 1:** o-Ps mean lifetime (τ_o-Ps_) and production intensity (I) in 3D melanoma spheroids without medium and chemical compounds.

Cell line	4th day	8th day
	τ_o-Ps_ [ns], I (%)	τ_o-Ps_ [ns], I (%)
WM266-4	1.876 (0.005), 16.5% (0.6)	1.861 (0.006), 17.0% (0.2)
WM115	1.909 (0.006), 18.0% (0.8)	1.875 (0.006), 15.0% (0.2)

## Discussion

Recently, positronium imaging was introduced^14,15,19,46^, and the first in vitro positronium images were demonstrated^25,26^, opening new possibilities for the improvement of cancer diagnosis by using positronium as a tissue pathology biomarker^13,14^.

The main aim of this study was to test the hypothesis that positronium may serve as a novel biomarker for assessing the different cancer activities and biological properties in cancer cells and to perform the first studies of positronium properties in 3D cell spheroids. To test this hypothesis, 3D spheroids were used because they can mimic the structure of real tumors under physiological conditions. The morphology and cell–cell interactions in 3D spheroids are completely different than those in monolayer cell culture. Moreover, they hold certain advantages over standard research methods, including low cost of use, high reproducibility, timesaving, and reduced need for laboratory animal models. The unique properties of 3D tumor spheroids make them invaluable for biological experiments and drug tests in a variety of experimental studies focused on chemotherapy and radiotherapy^1–5,47,48^.

The cell cycle time in 2D cells and 3D spheroids is significantly different because cells in monolayer cell culture have homogenous biological conditions; in a confluent monolayer, most of the cells are in the same cell cycle and have sufficient accessibility to nutrients and oxygen. In 3D cell culture with a multilayer structure, cells are in different cell cycles, and accessibility to necessary nutrients for survival depends on their distance from the surface^49^. During the cell cycle, cells in the G1 phase grow up to the end of the G2 phase when mitosis and division start after passing the checkpoints. In spheroids, space for proliferation is limited, and a necrotic core is formed surrounded by a quiescent layer where cells are arrested in the G1 phase of the cycle, and a proliferating rim at the outermost part of the spheroids where cells can stay in the S/G2/M phase^50^. A significant difference in the dynamics of spheroid formation and cell proliferation was observed in our study. We observed that the more malignant cell line (WM266-4) formed larger spheroids (both on days 4 and 8 in culture) with a higher number of cells (Fig. 5B,C), and the cell diameter in the spheroids was lower than that in the primary melanoma cell line (WM115).

Prior positronium lifetime research has been mostly performed on different types of tissues and monolayer cell cultures, such as skin cancer and colorectal cancer cells^20–23,46,51,52^. In this novel research, the lifetime of positronium was evaluated in 3D spheroids rather than 2D cell culture or tissue. Although much research has been performed on positronium in materials and biological samples, research using 3D cell aggregates is limited to the determination of the *o*-Ps lifetime in 3D colorectal cancer cell aggregates with a mixture of cells and collagen^20^. The *o*-Ps mean life time was shown to be a sensitive indicator of mean void volume in 3D cancer cultures stimulated with the growing factor affecting epithelial cell stimulation (TGF-β). Colonies treated with TGF-β showed growth with the mean *o*-Ps 2–3 weeks after stimulation, which decreased to the value under control conditions in the late phase of culture^19^. This *o*-Ps distribution indicated a possible relationship between cell dynamics and mean molecular voids. The diffusion behavior of small molecules (*Å*) has been investigated using PALS in different biological samples (hair, feather, cotton and silk), showing that *o*-Ps are sensitive to void investigation in biological polymers^53,54^. The lifetimes of *o*-Ps in the cell can be different from the lifetime measured in the case when the cell is in the medium containing water, collagen, or other chemical compounds because the cell surface, cell growth, and the shape of cells can be changed.

In our study, we focused on the early stage of spontaneous proliferation of two different cancer cell lines. These cell lines differ in cell size, with WM155 cells being larger than WM266-4 cells. Thus, during spheroid formation, WM266-4 cells compensate for their smaller size with a higher rate of proliferation to form larger spheroids. Their doubling time was shorter than that of WM115 cells, and WM266-4 cells spent less time in their cell cycle than WM115 cells. These important differences in the cell dynamics in the early stage of spheroid growth were indicated by the shorter *o*-Ps lifetime for the WM266-4 spheroids on day 4 of culture. In our previous study, we compared the percentage of viable cells as assessed by the FDA/PI fluorescent assay (fluorescein acetate and propidium iodide) in situ, and we did not observe any difference between cell viability in the early phase of spheroid formation (on day 4) for these two melanoma cell lines, but a larger necrotic core was recognized in the WM115 spheroids in comparison to WM266-4 (29.36 vs. 13.66% of dead cells)^38^. These results are in concordance with the glucose and hypoxia distribution assays, where the core of the spheroid was recognized as the region with the lowest glucose concentration and the highest hypoxic conditions. Decreased glucose concentration (possibly caused by reduced diffusion or increased anaerobic metabolism—glycolysis) was observed in older spheroids (day 8 in culture) and those formed from more cells (Fig. 6). Interestingly, the irregular shape of WM115 allowed the fluorescent probe to penetrate deeper into the core, which was observed as a peak in fluorescence intensity in the region between 50 and 75 µm from the center of the spheroid (Fig. 6D). This glucose availability may facilitate anaerobic glucose metabolism (glycolysis) in the WM115 cell line and may be related to the higher *o*-Ps mean life time in these conditions.

**Figure 9:**
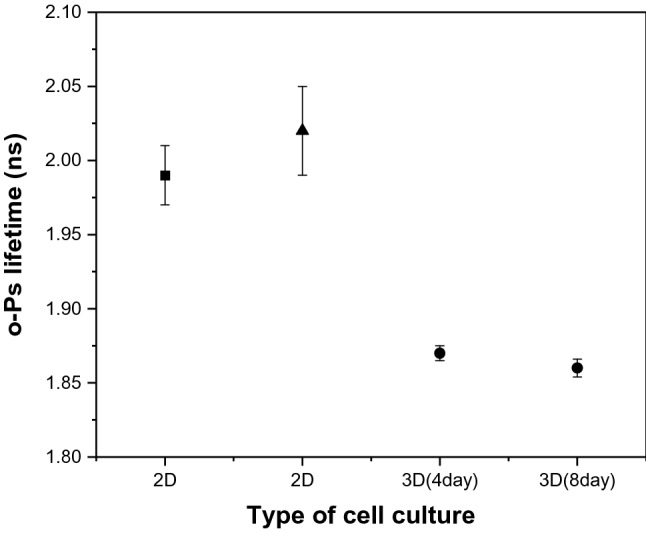
Comparison of mean positronium lifetime in 2D and 3D cell culture of melanoma. The lifetime of *o*-Ps in WM266-4 2D cell culture (black square) according to Ref.^55^, the lifetime of *o*-Ps in WM266-4 2D cell culture (black triangle), and the lifetime of *o*-Ps in WM266-4 3D cell culture (black dots) show the *o*-Ps lifetime in spheroids of two different ages, 4 and 8 days after cell seeding.

We also performed additional measurements to compare the *o*-Ps lifetime in 2D and 3D cell cultures. The obtained results (Fig. 9) demonstrate that the positronium lifetime is smaller in 3D cell culture than in 2D cell culture^55^. This graph shows how the mean *o*-Ps lifetime is different between 2 and 3D cell cultures, which is due to their diversity in biological properties and metabolism. The WM155 and WM266-4 cell lines were characterized by different gene expression, e.g., those responsible for amino acid transport, and many others that are related to the formation of cell junctions, migration and mobility^12,56^.

This study was conducted to evaluate the lifetime distribution of ortho-positronium in WM266-4 and WM115 melanoma cell spheroids characterized by different degrees of malignancy. The measurements were performed at two different times (4 and 8 days) after seeding. During this time, both spheroid cell lines showed a reduction in *o*-Ps lifetime, which backs to the gradual cell proliferation in spheroids. More malignant WM266-4 spheroids showed a shorter *o*-Ps lifetime than WM115 cell line spheroids.

In summary, the results show that the lifetime of *o*-Ps is a useful parameter to differentiate between cancer cells with different biological characteristics. The higher the degree of malignancy and the rate of proliferation of neoplastic cells, the shorter the lifetime of ortho-positronium. This research paves the way for the development of positronium biomarkers using a cell spheroid model.

## Supplementary Information

Supplementary Information.

## Data Availability

The datasets generated and analysed during the current study containing numerical data, mathematical models, and calculation results, including fitting graphs, are available in the Jagiellonian University Repository (RUJ) at the link https://ruj.uj.edu.pl/xmlui/handle/item/297405^57^.
