# Supplementary material for: 3D melanoma spheroid model for the development of positronium biomarkers
Source: Sci Rep. 2023 May 11;13:7648. doi: 10.1038/s41598-023-34571-4 (PMC10175546; doi:10.1038/s41598-023-34571-4)
Supplement: Supplementary file 1 — Supplementary Information. [file 41598_2023_34571_MOESM1_ESM.pdf]

Supplementary File 1

3D melanoma spheroid model for the development of positronium biomarkers

Hanieh Karimi [a,b], Paweł Moskal[c,d], Agata Żak[e], Ewa Ł. Stępień [a,d*]

[a] Department of Medical Physics, M. Smoluchowski Institute of Physics, Faculty of Physics, Astronomy and Applied Computer Science, Jagiellonian University, Kraków, Poland

[b] Department of Biochemistry, University of Missouri, Columbia, United States

[c] Department of Experimental Particle Physics and Applications, M. Smoluchowski Institute of Physics, Faculty of Physics, Astronomy and Applied Computer Science, Jagiellonian University, Kraków, Poland

[d] Center for Theranostics, Jagiellonian University, Kraków, Poland

[e] Faculty of Chemistry, Jagiellonian University, Kraków, Poland

*To whom correspondence should be addressed

Prof. Ewa Ł. Stępień
Department of Medical Physics
Faculty of Physics, Astronomy and Applied Computer Science
Jagiellonian University
Łojasiewicza 11 Street
30-348 Kraków
e-mail: e.stepien@uj.edu.pl
tel. +48 12 664 4762

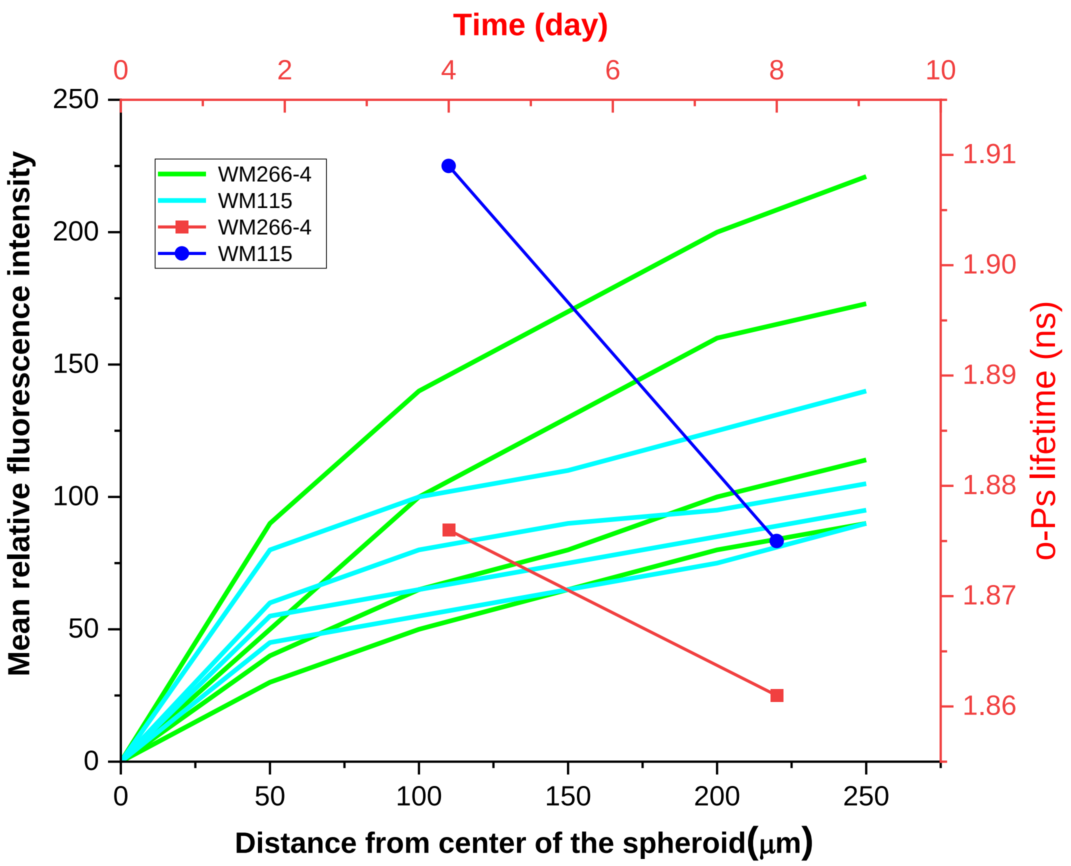

Supplementary Figure 1. Comparison of the mean o-Ps lifetime and intensity for different melanoma cell lines in relation to spheroid metabolism.

The lifetime of o-Ps in the WM266-4 (blue dot) and WM115 (red square) spheroids on two different days (4 and 8) after cell seeding compared with the hypoxia distribution in the spheroids represented as the distance from the spheroid center in WM266-4 (gren line) and WM115 (turqoised line).

Oxygen uptake rate distribution was determined using a hypoxia kit, Image-IT™ Green Hypoxia Reagent (Thermo Fisher Scientific, cat. no I14834). To assess the oxygen uptake rate distribution in the spheroids, WM266-4 and WM115 cells were seeded at densities of 1000 and 2000 cells/drop. On days 4 and 8, the spheroids were transferred to glass-bottom dishes, and 20 μl of Image-IT™ Hypoxia Reagent (10 μM) was added to each spheroid. The spheroids were incubated at 37°C for 1 hour. The hypoxia dye was exchanged with fresh growth medium, and the spheroids were placed again in a cell culture incubator for the next 4 hours. Spheroid images were taken with a Nikon Eclipse Ti-E microscope coupled to an A1 scanning confocal system (Nikon, Japan) at a 488 nm/520 nm fluorescent wavelength.
